# Effect of shRNA Mediated Silencing of YB-1 Protein on the Expression of Matrix Collagenases in Malignant Melanoma Cell In Vitro

**DOI:** 10.3390/cells7010007

**Published:** 2018-01-10

**Authors:** Wisam Nabeel Ibrahim, Abd Almonem Doolaanea, Mohammad Syaiful Bahari Bin Abdull Rasad

**Affiliations:** 1 Department of Basic Medical Sciences for Nursing, Faculty of Nursing, International Islamic University Malaysia, Kuantan Pahang 25200, Malaysia; 2 Department of Pharmaceutical Technology, Faculty of Pharmacy, International Islamic University Malaysia, Kuantan Pahang 25200, Malaysia; monem@iium.edu.my; 3 Department of BioMedical Sciences, Faculty of Allied Health Sciences, International Islamic University Malaysia, Kuantan Pahang 25200, Malaysia; syaiful@iium.edu.my

**Keywords:** YB-1, MMPs, collagenases, shRNA, malignant melanoma

## Abstract

*Background and Objective:* YB-1 is a transcription and oncogenic factor capable of binding to DNA and RNA performing versatile functions within normal and cancer cells. Some studies reported the binding of YB-1 with a collagenases gene promoter and influencing their expression. In addition, the role of YB-1 in malignant melanoma was not elucidated. Thus, in this study, the aim was to knock down the expression of YB-1 in A375 malignant melanoma cancer cell using the shRNA approach and study its effect on cancer cell proliferation, migration, and expression of collagenases. *Methods:* A375 malignant melanoma cell lines were grown in standard conditions and were transfected with three plasmids containing a retroviral pGFP-V-RS vector, two of them containing targeting sequences for YB-1 mRNA. The third plasmid contained a scrambled mRNA sequence as a negative control. Expression of YB-1 was validated using immune-fluorescence staining, RT-PCR and western blotting. The cancer cell proliferation was determined using MTT assay, serial trypan blue cell counting and cell cycle flow-cytometry analysis. Expression of collagenases (MMP1, MMP8, and MMP13) was evaluated using RT-PCR and western blotting analysis. In addition, a wound-healing assay was used to assess cell migration potential. Statistical analysis was performed using one-way ANOVA test with Bonferroni post hoc analysis to compare the quantitative results among samples. *Results:* The established silenced cell strains (P1 and P2) had nearly 70% knockdown in the expression of YB-1. These YB-1 silenced strains had a significant cell cycle-specific reduction in cell proliferation (*p* < 0.05 in serial cell counting and cell cycle flow cytometry analysis, *p* < 0.001 in MTT assay). In addition, YB-1 silenced strains had a remarkable reduction in cell migration potential. Expression of MMP13 was significantly reduced in YB-1 silenced strains. *Conclusion:* YB-1 oncoprotein is a promising target in the treatment of malignant melanoma. Silencing of this protein is associated with significant anti-proliferative, anti-invasive and MMP13 insulating properties in A375 malignant melanoma cancer cell lines.

## 1. Introduction

Malignant melanoma is a neoplasm of melanocytes. It is the third most common malignant skin cancer and is the most serious cancer regarding local invasiveness and mortality rate [1]. Among the different stages of tumor progression, the metastatic type has the worse prognosis and is one of the most aggressive and drug-resistant cancers, with a median survival time of 6 months and a 5-year survival rate of less than 5% [2]. In general, the process of tumor cell invasion starts through the detachment of the cancer cells and proteolysis of the basement membranes by a group of extracellular matrix proteases that are known as the matrix metalloproteinases (MMPs) family of enzymes [3,4].

Collagenases enzymes are a subgroup of the MMPs family of proteases capable of degrading native fibrillary collagens (type I, II, III, V and IX) [5]. This subgroup of enzymes includes MMP1, MMP8, and MMP13 also known as collagenases-1, -2 and -3 respectively [6]. These enzymes are normally expressed at minimal tissue level to regulate important biological functions such as angiogenesis, bone development and wound healing [6,7]. Interests in these enzymes have grown considerably in cancer research because of their direct contribution in the degradation of extracellular matrix (ECM) components when expressed in high levels [8,9]. MMP1 is interstitial collagenases, initially, detected in highly invasive malignant melanoma together with MMP13 [3]. High expression of these MMPs has been implicated in the promotion of the metastatic potential of many types of cancer cells [9,10,11,12]. While the role of MMP8 has provoked some controversy in which some studies reported its effect on cytokine release in the cancer microenvironment in breast cancer and some ovarian cancer cell lines [13,14], other reports have shown that MMP8 possess some antitumor properties [10].

The expression of these collagenases is tightly regulated at transcriptional level by the existence of several *cis*-elements within the gene promoter sequence [5]. MMP1 and MMP13 share many *cis*-elements within their gene promoter and are therefore usually co-expressed or co-repressed within the context of the special tissue function [6]. MMPs with different *cis*-elements structure in their promoters will have a different expression pattern such as MMP8 gene promoter which is considerably different from MMP1 and MMP13 gene, containing the Sp-1 transcription factor binding site and lacks the AP-1 and AP-2 promoter sequence [6]. In pathological conditions, the expression level of these MMPs changes mainly due to activation and binding of multiple transcription factors to specific MMPs gene promoter sequence causing significant changes in the expression levels of respective MMPs [6,7]. Among these transcription factors, YB-1 (Y-box-binding protein 1) commands a special concern in cancer progression. This multifunctional protein belongs to the cold-shock protein family, which is characterized by having a highly conserved nucleic-acid-binding motif binding to both DNA and RNA [15,16]. This enables YB-1 to have versatile roles in regulating cellular DNA repair, RNA splicing, exon skipping [15,16]. However, YB-1 is also considered as an oncoprotein because it is found to enhance uncontrolled proliferation, the evasion from immune recognition and growth suppression, cancer cell immortality, sustained angiogenesis, invasion, and metastasis in several malignancies [15,16].

Accordingly, elevated levels of YB-1 protein were highly correlated with cancer progression and poor prognosis in many malignancies such as breast cancer, lung cancer, osteosarcoma, melanoma and multiple myeloma [15]. The relationship between YB-1 protein and the expression of collagenases MMPs, in general, is not well elucidated; however, some studies had reported some interference among them [17,18,19]. YB-1 was reported to have an insulating effect on the expression of MMP13 through interaction with the AP-1 sequence of the gene promoter [20]. However, this interaction was contradicted in the previous study [21]. Therefore, the effect of YB-1 in regulating the expression of collagenases MMPs is yet poorly revealed. This study aimed to establish a stable A375 cell line with constant knockdown of YB-1 protein and to investigate its effect on the expression of collagenases MMPs, in addition to the exploration of other anti-tumor properties.

## 2. Results

### 2.1. YB-1 Silencing via shRNA Mediated RNA Interference in A375 Malignant Melanoma Cell Line

**Figure 1:**
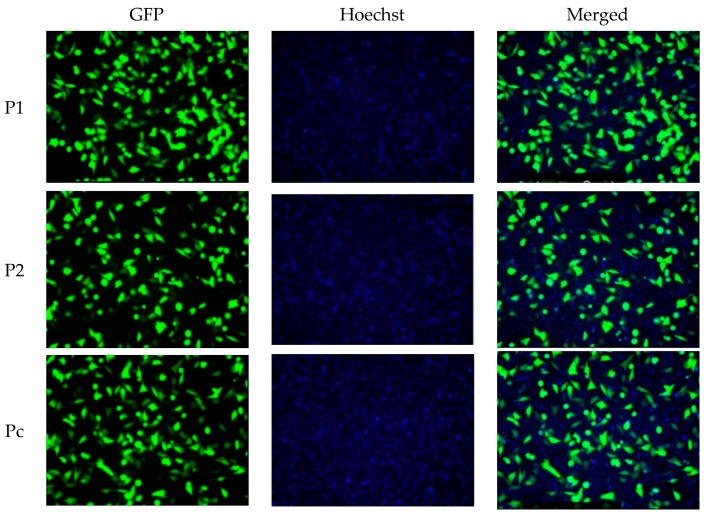
Transfection of A375 cells. A375 cells were transfected with two YB-1 silencing shRNA plasmids (P1 and P2) with one nontargeting shRNA plasmid (Pc) as a negative control. All plasmids were containing a pGFP-V-RS vector that emits GFP fluorescent light upon successful expression of each plasmid. Images were captured Cytell™ Cell Imaging System.

**Figure 2:**
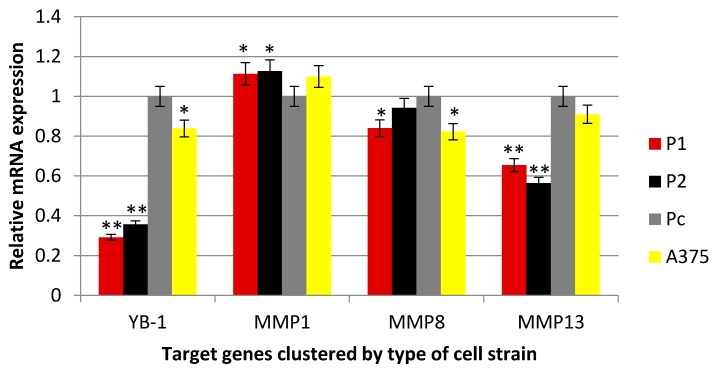
Real-time PCR analyses.YB-1 and matrix collagenases (MMP1, MMP8, and MMP13) gene expression analysis to detect the relative mRNA expression of each gene in the transfected cells with the comparison with parent A375 cell line level. The Ct values were presented as mean ± standard error and were normalized first with internal control genes. One way ANOVA test was used to analyze the data, * *p* < 0.05, ** *p* < 0.001.

**Figure 3:**
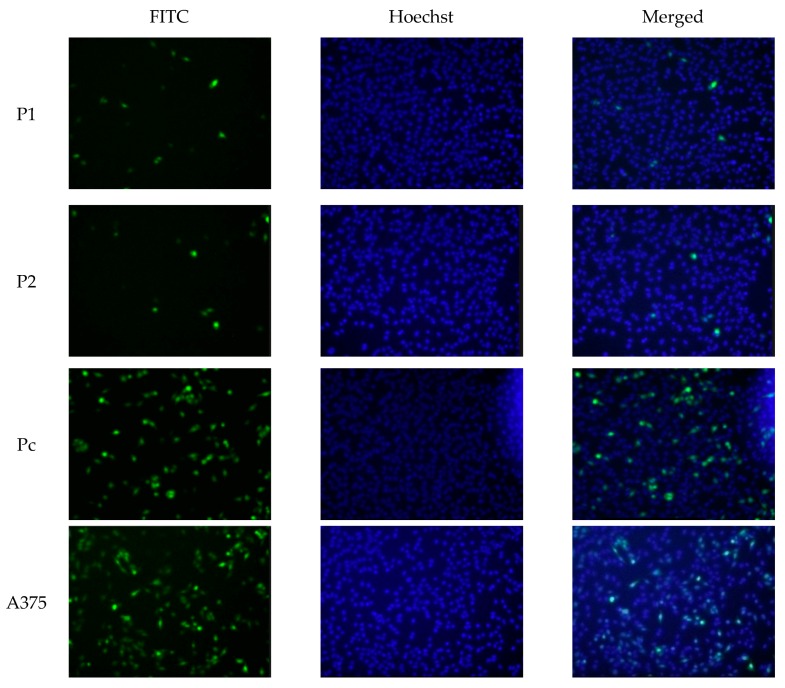
Immune fluorescence staining. YB-1 knockdown was validated using primary mouse anti YB-1 monoclonal antibodies and secondary goat anti-Mouse IgG antibodies tagged with green FITC fluorescent stain. The nontoxic Hoechst nuclear staining was used as well. The low expression levels of YB-1 is confirmed in P1 and P2 cell strains while higher expression levels were detected in Pc cell strain and the parent A375 cell line.

**Figure 4:**
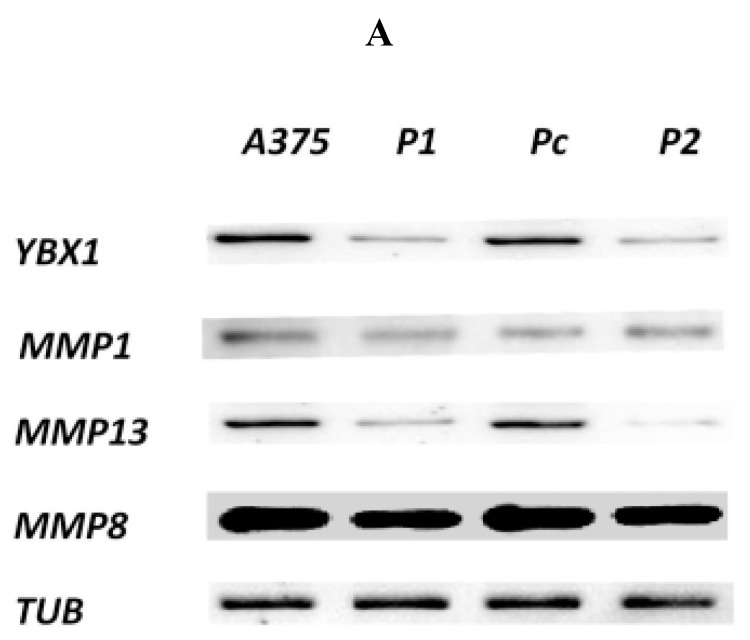

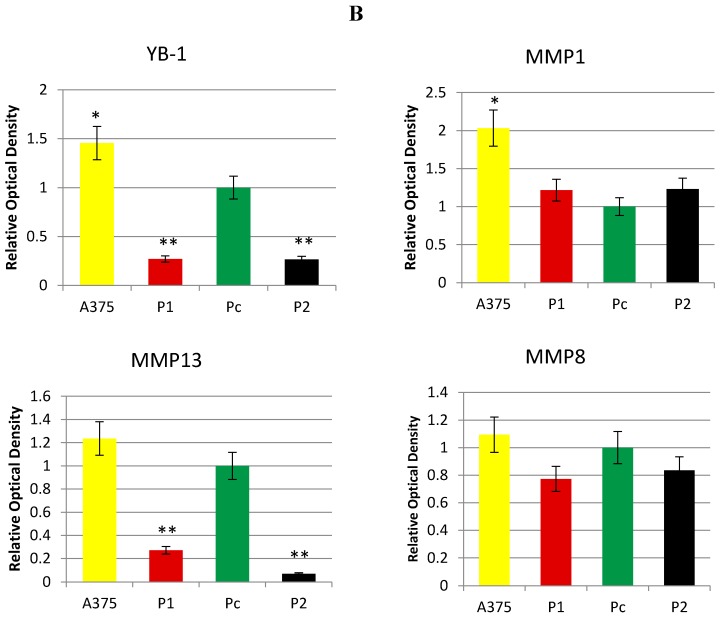
Western blot and densitometry analysis. (**A**) Expression levels of target proteins were assessed by western blotting with alpha-tubulin as an internal control in the selected cell strains. The molecular weight was approximate as follows (MMP1: 54 kDa, MMP8: 53 kDa, MMP13: 54 kDa, YB-1: 45 kDa and TUB: 50 kDa); (**B**) Densitometry analysis by imagJ the quantitative results were expressed as means ± standard error compared with Pc cell strain and analyzed using one-way ANOVA, * *p* < 0.05, ** *p* < 0.001.

In the current study, the two shRNA plasmid constructs were cloned into a pGFP-V-RS vector (OriGene, Rockville, MD, USA). The transfection was validated by green fluorescent protein light detection by Cytell™ Cell Imaging System as shown in Figure 1. Hoechst nuclear staining was used to stain the nuclei without interfering with the viability of the different cell clones. The level of YB-1 mRNA (Figure 2) and protein (Figure 3 and Figure 4) was successfully knocked down in P1, and P2 cell strains in comparison with Pc and the parent A375 cell strains. This established an A375 cell strain with a permanent, stable knockdown of YB-1 protein.

### 2.2. Antiproliferative Effect of YB-1 Silencing in A375 Cell Line

**Figure 5:**
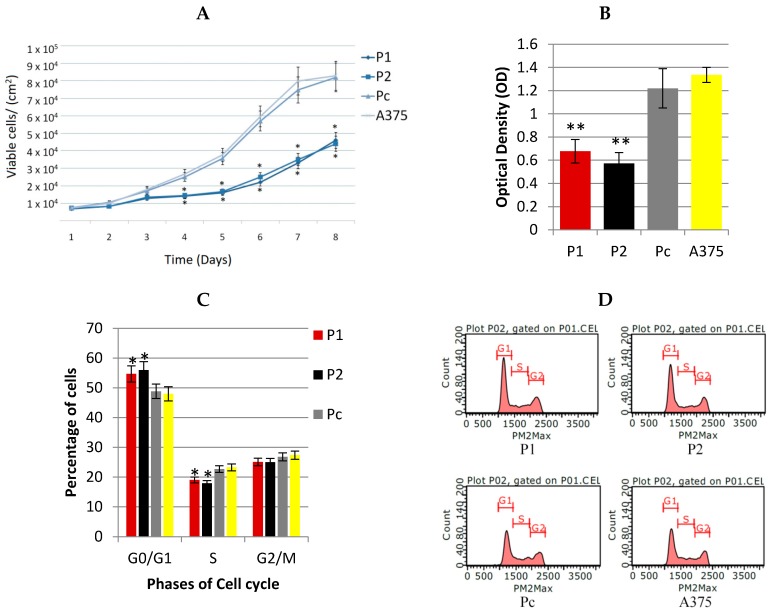
Anti-proliferative effects of YB-1 shRNA (**A**) Colorimetric MTT assay performed by measuring the value of optical density at a wavelength of 590 nm with a reference filter of 620 nm by TECAN Infiniti plate reader; (**B**) Serial cell counting for different cell strains to detect the pattern of exponential cell growth by trypan blue stain; (**C**,**D**) Flow cytometry cell cycle analysis of the different cell strains to detect any interference by YB-1 shRNA by Guava easyCyte flow-cytometer. All the quantitative results were presented as means ± standard error compared with Pc cell strain and analyzed using one-way ANOVA, * *p* < 0.05, ** *p* < 0.001.

In this study, the serial cell counting has shown a significant (*p* < 0.05) reduction in cancer cell proliferation among P1 and P2 YB-1 silenced cell strains in comparison with Pc cell strain as shown in Figure 5A. The MTT results were compatible with the cell counting findings, showing a highly significant reduction in the optical density among P1 and P2 YB-1 silenced cell strains in comparison with Pc cell strain as shown in Figure 5B. Moreover, the flow-cytometry results have shown YB-1 as a cell cycle specific regulator of cell proliferation as shown in Figure 5C,D. There was a significant accumulation of cancer cells within the G0/G1 phase among the YB-1 silenced cell strains (P1 and P2, (*p* < 0.05)) in comparison with Pc cancer cell strains. The cell cycle arrest in G0/G1 possibly explains the role of YB-1 oncogenic factor in A375 malignant melanoma cancer cell proliferation.

### 2.3. Effect of YB-1 Silencing on Expression of Matrix Collagenases in A375 Cell Line

In this study, MMP13 gene was significantly underexpressed in P1 and P2 YB-1 silenced cell strains (*p* < 0.05) in comparison with Pc cell strain as shown in Figure 3, while the expression of MMP1 and MMP8 genes were not significantly different among the cell samples (*p* > 0.05). The protein expression demonstrated by the western blotting analysis was consistent with changes in mRNA levels whereby, the P1 and P2 YB-1 silenced cell strains had a significantly lower expression of the MMP-13 enzyme (*p* < 0.05). The changes in MMP1 and MMP8 enzymes were also not significantly different from the Pc control sample (*p* > 0.05) as shown in Figure 4.

### 2.4. Effect of YB-1 Silencing on A375 Cell Line Migration Assay In Vitro

**Figure 6:**
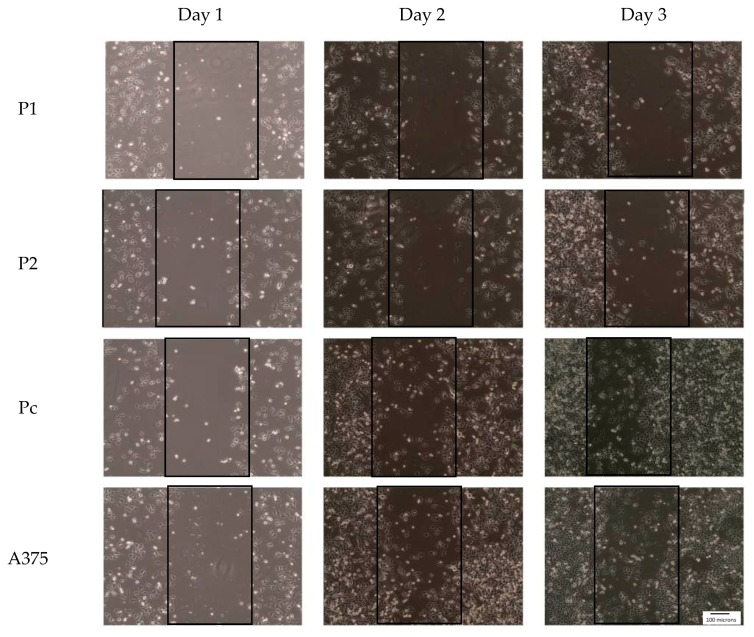
Effect of YB-1 shRNA on cancer cell migration (wound closure assay): cells were visualized with 10× magnification power under a light microscope. The wounds were monitored every 24 h for three days to determine the rate of closure by the migratory cancer cells.

In the study, the established YB-1 silenced cell strains (P1 and P2) had a significantly less migration pattern in comparison with control cell strain (Pc) as demonstrated in Figure 6. On day 3 of the wound closure assay each of the cancer cell strains were assessed under the microscope for migration potential that was clearly more prominent in the control cell strain and very minimal in the YB-1 silenced cell strains although the proliferation of control samples were significantly higher and could participate in the cell migration process as shown in the figure.

## 3. Discussion

The knockdown of YB-1 protein in this study has shown some promising properties by reducing the proliferation of cancer cells, interference with cancer cell cycle, inhibiting the cancer cell migration in vitro; in addition to the influence on MMP13 expression. This approach was also validated in other types of cancers where YB-1 RNA interference has shown promising results in neuroblastoma [22], leukemia [23] and glioblastoma [24] tumors.

### 3.1. YB-1 Protein and A375 Cancer Cell Proliferation

In this study, the YB-1 protein showed a cell cycle specific role in regulating the proliferation of A375 cancer cells. The percentage of cells in G1/G0 phase was significantly higher among the YB-1 knocked down A375 cell strains (P1 and P2) in comparison with the control Pc strain. In addition, the proliferation of these knocked down strains was considerably lower than the control strain as demonstrated in the MTT assay and the serial cell counting shown in Figure 5A–D. This inhibitory effect of YB-1 knockdown was demonstrated in vitro and in vivo in many types of malignancies including breast, colon, lung and skin cancers [15,25]. This effect was also demonstrated through interference with phases of cancer cell cycle [22,24]. This effect could be explained by the versatile binding specificity of YB-1 to the promoters of pro-growth genes enhancing their expression. However, studies have attempted to predict the specific binding site of YB-1 involved with cancer cell proliferation and had concluded that the sequence is dependent on cancer cell type and on the presence of YB-1 co-factor that could alter YB-1 binding specificity [26] which adds more ambiguity to roles played by YB-1 protein in this regard. Another possible contributor in the cell cycle arrest in this study is MMP13. Because the YB-1 silenced cell strains also had a knockdown of MMP13 expression, this role was supported by another study done on A375 melanoma cells, where RNAi of MMP13 resulted in a similar cell cycle arrest in G1/G0. In addition, MMP13 may generate autocrine loops that contribute to activation of growth factors receptors. However, the detailed mechanism of MMP13 within the cell cycle of melanoma cancer cells was not revealed. Instead, a postulation was constructed assuming that MMP13 may play a role in cleaving cell bound inactive growth factors such as heparin binding epidermal growth factor (HB-EGF) or transforming growth factor alpha (TGF-α) [27].

### 3.2. YB-1 Protein and the Expression of Collagenases MMPs in A375 Cancer Cells

In this study, YB-1 silenced A375 cell strains (P1 and P2) had a significant reduction in the expression of MMP13. This effect was contradicted in a previous study conducted by Samuel [20], in which YB-1 protein was shown to interact with the AP-1 sequence of MMP13 gene promoter insulating its expression in HeLa cells. However, this insulating effect was not confirmed in malignant melanoma cells in the previous and current study [21]. There is no additional evidence reporting the influence of YB-1 on the expression of collagenases. However, the relationship with other MMPs members has been elucidated by the knockdown of YB-1 expression in malignant melanoma cancer cells, which did show a reduction in the expression of MMP2 and MMP14 [18,19]. Additionally, the study conducted by Sarkar et al. [17] demonstrated that YB-1 protein directly enhanced the expression of MMP-2 by interaction with the specific gene promoter sequence. The YB-1 protein may regulate the expression of MMPs by directly binding to specific gene promoter sequences. However, it is challenging to identify these specific sites, which largely depend on the cellular context and presence of other co-partners, which determine the final binding sequence [15]. YB-1 may also indirectly affect MMPs gene expression by enhancing the translation of other transcription factors such as the signal transducers and activators of transcription (STAT), nuclear factor kappa B (NF-κB), the Smad family of proteins, and the mitogen-activated protein kinases (MAPK) [16]. Additionally, the YB-1 protein may regulate the function of MMPs by controlling the expression of other proteases capable of activating pro-MMPs such as plasmin enzyme [26].

Therefore, identification of other co-partners (transcription factors) or novel binding sequences within MMP13 gene promoter is crucial to determine the mechanism of YB-1 interference with collagenases MMPs expression in melanoma cells.

### 3.3. YB-1 Protein and A375 Cancer Cell Migration

Yb-1 protein knock down through shRNA interference was associated with obvious reduction in melanoma cancer migration behavior. This effect might be attributed to the deactivation of cadherin molecules [15]. Another possible mechanism is by induction of epithelial-mesenchymal transformation (EMT) [28]. This might explain the invasive potential of cells with highly expressed YB-1 protein [29].

## 4. Materials and Methods

### 4.1. Establishing the Stable YB-1 Silenced Malignant Melanoma Cell Line

The A375 malignant melanoma cell line was purchased from American Type Culture Collection ATCC (Manassas, VA, USA). The cells were cultured in Dulbecco’s Modified Eagle’s medium (DMEM) (Gibco, Carlsbad, CA, USA) supplemented with 10% fetal bovine serum (FBS) (Gibco), 1 mM sodium pyruvate (Gibco), 10 mM 4-(2-hydroxyethyl)-1-piperazineethanesulfonic acid (HEPES) buffer (Gibco). The cells were maintained in a humidified incubator at 37 °C with 5% CO_2_. To establish the stable YB-1 silenced A375 cell line, two short hairpin RNA (shRNA) plasmid constructs cloned into retroviral pGFP-V-RS vector (OriGene, Rockville, MD, USA) were used with targeting sequences to YB-1 mRNA in the first plasmid (P1) as follows: 5′-TTCATCAACAGGAATGACACCAAGGAAGA-3′ and second plasmid (P2) as follows: 5′-GACAGCCTAGAGAGGACGGCAATGAAGAA-3′. In parallel, a non-targeting scrambled sequence plasmid construct in a retroviral pGFP-V-RS vector (OriGene) was used as a negative control (Pc).

The plasmids were amplified in JM109 competent cells (Promega, Madison, WI, USA) grown in Miller’s LB Broth (Sigma-Aldrich, Steinheim am Albuch, Germany) supplemented with 25 µg/mL of Kanamycin sulfate (Sigma-Aldrich). Then the plasmids were extracted using PureYield™ Plasmid Miniprep kit (Promega) followed by plasmid quantification and qualification by estimating the A260/A280 and the A260/A230 ratios using Nanodrop spectrophotometer (Thermo Scientific, Waltham, MA, USA) and 1% agarose gel electrophoresis.

The A375 cells were grown in 24 well plates. After reaching 75% confluence, the transfection was performed with ViafectTM Transfection kit (Promega) following the manufacturer instructions by mixing 1 µg of the plasmid in 100 µL of serum-free media with 4 µL of transfection reagent. The cells were then incubated for two days before validating the transfection by analyzing the GFP reporter gene function in A375 cells in each well using Cytell™ Cell Imaging System (GE Healthcare, Marlborough, MA, USA). After confirmation of GFP fluorescence as shown in Figure 1, the cells were then cultured by changing the media every three days with complete media containing (1 µg/mL)and (0.5 µg/mL) of puromycin in alternate weeks for one month during which stable clones were selected and passaged for later experiments. The expression of YB-1 was determined in the selected clones using real-time PCR, immunofluorescence and immunoblotting analysis.

### 4.2. Real-Time PCR

The untreated A375 cells, P1, P2 and Pc clones were maintained in complete non-selective media till reaching 70% confluence in 25 cm^3^ cell culture flasks. Then the complete media was replaced with serum-free media for 24 h followed by detaching the cells using triple E-express (Gibco, Carlsbad, CA, USA). The cells were washed twice with Phosphate-buffered saline (PBS) (Thermo Fisher, Waltham, MA, USA) (pH 7.4) followed by centrifugation and mRNA extraction using AurumTMTotal RNA Mini kit (BioRad, Hercules, CA, USA) following the manufacturer protocol. Before proceeding, the RNA integrity was tested on a 1% denaturing agarose gel stained with ethidium bromide by visualizing the 18S and 28S bands with no evident smearing in addition to Nanodrop spectrophotometer determination of RNA purity (Thermo Scientific, Waltham, MA, USA) by assessing the A260/A280 and the A260/A230 ratios being higher than 2.00. The RNA samples were then normalized with RNA elution buffer based upon the Nanodrop measures. The iScript™ Advanced cDNA Synthesis Kit (BioRad, Hercules, CA, USA) was used to synthesize cDNA from each of the mRNA samples following the manufacturer protocol. Each of cDNA samples was then subjected to real-time PCR using SsoAdvUniver SYBR Green master mix (BioRad, Hercules, CA, USA) with specific primer sequences for YB-1, MMP1, MMP8 and MMP13 (BioRad, Hercules, CA, USA). After completion of amplification, the Ct values of each well was determined and used for relative gene expression analysis following ΔΔCt method by normalizing the mean Ct values of target proteins with housekeeping genes including glyceraldehyde 3-phosphate dehydrogenase (GAPDH), beta-actin and alpha tubulin among the experimental and control samples to determine the fold difference in the relative mRNA expression of each protein. Each of samples experiments involved triplicate repeats with multiple replicates for validation of the results.

### 4.3. Protein Extraction and Western Blot Analysis

The four cell lines were treated similarly as in the real-time PCR cell whereby the cell samples were harvested and washed twice with PBS buffer followed by centrifugation each time. The cell pellets of different samples were mixed with 400 µL of Pierce RIPA lysis buffer (25 mM Tris-HCl (pH 7.6), 0.1% sodium dodecyl sulfate (SDS), % sodium deoxycholate, 150 mM NaCl, 1% NP-40) (Thermo Fisher, Waltham, MA, USA) on ice by pipetting and vortexing. The mixtures were also subjected to water bath sonication for 30 s every 10 min while keeping the protein samples on ice for 30 min. Each of the protein lysates was then centrifugated at 12,000 RPM for 30 min at 4 °C followed by careful aspiration of the protein lysate solution without aspirating the precipitate in the bottom of the tube. The protein lysates concentration was determined in each sample using the colorimetric Pierce bicinchoninic acid (BCA) assay kit (Thermo Fisher, Waltham, MA, USA) following the standard protocol to plot the standard curve using the standard protein solutions followed by quantification of total protein in each of the experimental and control samples. 15 µg of protein in Laemmli Sample Buffer (BioRad, Hercules, CA, USA) was loaded into each lane of the mini proteon 12% TGX free SDS-polyacrylamide gel (BioRad, Hercules, CA, USA) followed by electrophoresis for 90 min at 100 V. After confirming the presence of protein bands in the gels, the proteins were blotted to 0.45 µm pore size Polyvinylidene fluoride (PVDF) membrane using Trans-Blot^®^ SD Semi-Dry Transfer Cell (BioRad) at 18 Volt for 45 min. After confirming the presence of protein bands in the membranes, the membranes were then blocked with Blocking One solution (Nacalai, Nakagyo-ku, Kyoto, Japan) for 30 min followed by overnight incubation with primary specific monoclonal mouse antibodies (Santa Cruz Biotechnology, Dallas, TX, USA) at 4 °C with aggression. Then the membranes were washed three to five times with PBS buffer including 0.1% Tween 20 (PBST) buffer (pH 7.4) and were then incubated with horseradish peroxidase (HRP)-conjugated secondary Rabbit anti-mouse antibodies solutions (Santa-Cruz, Germany) for 1 h at room temperature. After five times of more aggressive washing, Luminata Forte Western HRP substrate (Millipore, Burlington, MA, USA) was added to the membrane, and the protein bands were detected and analyzed using ChemiDoc™ XRS (BioRad, Hercules, CA, USA).

Densitometric analysis of the western blotting results was performed using ImageJ software (NIH, Bethesda, MD, USA) [30].

### 4.4. MTT Assay and Cell Counting

The proliferative potential of cell samples was determined using 3-(4,5-Dimethylthiazol-2-Yl)-2,5-Diphenyltetrazolium Bromide (MTT) assay and conventional cell counting. In MTT colorimetric assay, approximately 7.5 × 10^3^ cells were plated to each well of 96 well plate in 100 µL of complete media. Next day the complete media was replaced with 100 µL of serum-free media and kept for 24 h in the humidified CO_2_ incubator at 37 °C. The next day, the media was carefully removed from each well followed by washing with PBS buffer and adding 100 µL media containing MTT reagent (0.5 mg/mL). The cells were then incubated for 4 h in the in a humidified CO_2_ incubator at 37 °C, followed by aspirating the media containing MTT and adding 100 µL of dimethyl sulfoxide and the 96 well plate was covered with aluminum foil and agitated for two hours. Then the plate was read at 590 nm with a reference filter of 620 nm using TECAN Infiniti plate reader (TECAN, Männedorf, Switzerland).

Cell Viability = (experimental sample absorbance − blank sample absorbance)/(control sample absorbance − blank sample absorbance) × 100

In parallel, the proliferation of each cell sample was assessed by conventional cell counting with trypan blue staining.

### 4.5. Immuno-Fluorescence Staining

In this experiment, approximately 1 × 10^4^ cells from different cell samples were plated in 24 well plate with 500 µL of complete media and kept in the humidified CO_2_ incubator at 37 °C. The next day, the complete media was replaced with 500 µL of serum-free media and kept for 24 h in the humidified CO_2_ incubator at 37 °C followed by washing the wells twice with PBS buffer. The cells were then fixed with 4% formaldehyde in PBS buffer for one hour at room temperature followed by aspiration and washing with PBS buffer containing 5% FBS for three times. The fixed cells were then permeabilized with 0.1% Triton X-100 in PBS buffer solution containing 5% FBS for 20 min at room temperature followed by washing for three times with PBS buffer solution containing 5% FBS. The cell samples were then incubated with specific anti-YB-1 monoclonal mouse antibodies (Santa-Cruz, Germany) diluted in PBS buffer solution containing 5% FBS for one hour at room temperature, followed by aspiration and washing with PBS buffer solution containing 5% FBS for three times. Then the cell samples were incubated with secondary goat anti-Mouse IgG H&L (FITC) (Abcam, Cambridge, UK) antibody diluted in PBS buffer solution containing 5% FBS for one hour at room temperature followed by washing with PBS buffer solution containing 5% FBS for 3 times and the samples were then examined by Cytell™ Cell Imaging System (GE Healthcare, Marlborough, MA, USA).

### 4.6. Hoechst Nuclear Staining

Hoechst is a nuclear counterstain that permeates through the cell membrane and nuclear membrane without killing the cells to binds to DNA emitting a blue fluorescence stain. In the experiment, one drop of EasyProbes™ Hoechst 33342 Live Cell Stain (Genecopoeia, Rockville, MD, USA) was infused into each well of cell samples in the 24 well plate containing 500 µL of media. Then the plate was incubated at room temperature for 30 min followed by analyzing the plate with Cytell™ Cell Imaging System (GE Healthcare, Marlborough, MA, USA).

### 4.7. Flow Cytometry for Cell Cycle Interference

To determine the effect of YB-1 silencing on cell cycle, the DNA of different samples was stained with propidium iodide using Guava Cell Cycle Reagent (Millipore, Burlington, MA, USA). In this experiment, approximately 1 × 10^6^ cells from each cell sample were grown for 24 h in serum-free media and were then harvested and collected in 1 mL of media in 15 mL Falcon conical polypropylene tube. The cells were washed twice with PBS buffer solution and were centrifuged and resuspended in cold 70% ethanol and kept overnight at 4 °C. The cells were then washed twice with PBS buffer solution and resuspended and carefully mixed in dark environment with 600 µL of cell cycle reagent. 200 µL in triplicates from each sample was then transferred to each well in flat bottom 96 well plate and incubated in aluminum foil at 37 °C in a humidified incubator for 30 min followed by running the plate in Guava easyCyte flow-cytometer (Millipore, Burlington, MA, USA).

### 4.8. Wound Healing Assay

One of the valid methods to determine the invasion and cell migration in vitro is through creating a wound in the cells monolayer with subsequent evaluation of their migration towards the empty zone [31]. In this experiment, approximately 1× 10^5^ cells of the different cell strains were seeded to the wells of 24-well plate in triplicates and were grown in complete DMEM supplemented with 10% FBS. Upon reaching 60% confluence, the media was replaced with serum-free media for 24 h. The next day, a sagittal wound was created by gently scratching the monolayer cells across the center of the well in the plate by a 200 µL pipette tip followed by washing the wells twice with PBS buffer solution to remove the detached cells. After confirming the width of the wound, the cells were replenished with complete DMEM media supplemented with 10% FBS and were evaluated for cell migration.

### 4.9. Statistical Analysis

The statistical analysis was completed using SPSS 17.0 (SPSS Inc., Chicago, IL, USA). The samples numerical data were recorded as the mean ± standard error of the mean value, and the. Comparison between groups was made by one-way ANOVA test with Bonferroni post hoc test analysis. *p*-Values of less than 0.05 were considered statistically significant.

## 5. Conclusions

YB-1 protein is highly expressed by A375 malignant melanoma cell lines and is primarily responsible for the cancer cell proliferation, migration and mesenchymal transformation. In addition, YB-1 was shown to affect the expression of MMP13 in A375 cancer cell line without binding to the AP-1 binding site of MMP13 gene promoter. However, the specific binding site for YB-1 was not revealed in this study. Therefore, more effort is needed to investigate the effect of YB-1 silencing on different cancer signaling pathways and to identify novel gene binding sites and protein partners capable of interacting with YB-1 on these sites.
